# Genome assembly and annotation of *Rugonectria rugulosa*, a fungus associated with avocado (*Persea americana* cv. Hass) trunk canker in Colombia

**DOI:** 10.1128/mra.00512-26

**Published:** 2026-06-04

**Authors:** Juan Diego Rincón López, Katherine Chacón-Vargas, Diana Carolina Clavijo-Buriticá, Víctor Hugo García-Merchán

**Affiliations:** 1 Grupo de Investigación en Evolución, Ecología y Conservación (EECO), Facultad de Ciencias Básicas, Programa de Doctorado en Ciencias, Universidad del Quindío, Armenia, Colombia; 2 Synbiotic Health, Inc, Lincoln, Nebraska, USA; 3 Pontificia Universidad Javeriana, Facultad de Ingeniería y Ciencias, Instituto de Investigación iÓMICAS, Cali, Colombia; Nanchang University, Nanchang, Jiangxi, China

**Keywords:** *Rugonectria rugulosa*, genome assembly, fungal genomics, avocado, plant pathogen, Nectriaceae, secondary metabolites, trunk canker, wilting

## Abstract

*Rugonectria rugulosa* is a member of the family *Nectriaceae* associated with trunk canker diseases in woody plants, including avocado (*Persea americana* cv. Hass). Here, we report the 53 Mb draft genome assembly of a Colombian isolate, providing a genomic resource to investigate the biology and pathogenic potential of this species.

## ANNOUNCEMENT

*Rugonectria rugulosa* is a phytopathogenic fungus of the family *Nectriaceae* associated with woody plant diseases, such as stem cankers and wilt syndromes (1, 2). *R. rugulosa* has been reported as a trunk-associated pathogen causing dieback in forest trees, specifically *Falcataria moluccana* (3), and crops including *Prunus salicina* (4). Currently, woody diseases in avocado represent a major threat to production worldwide (5, 6). Thus, comprehensive genomic mapping of *R. rugulosa* is crucial to understanding its pathogenic mechanisms.

*R. rugulosa* strain M7C8 was isolated from active stem canker lesions of avocado cv. Hass trees collected in commercial orchards in Quindío, Colombia (N4°37′8″W75°38′9″), in 2024. Approximately 5 mm² symptomatic tissue fragments were surface-sterilized sequentially in ethanol 70%, sodium hypochlorite 1%, and sterile distilled water for 30 s each, air-dried at room temperature, plated on potato dextrose agar, and incubated at 28°C in darkness for 7 days (5). Fresh mycelium was collected for genomic DNA extraction using the DNeasy PowerSoil Pro Kit (Qiagen, Germany). Species identity was confirmed by PCR amplification and sequencing of the ITS1–5.8S–ITS2 region using primers ITS1/ITS4 (7). BLASTn searches showed 99.49% identity to the *R. rugulosa* strain BL1610.

Paired-end genomic libraries were prepared using the TruSeq Nano DNA Library Prep Kit (Illumina) with ~350 bp inserts and sequenced on the Illumina NovaSeq platform, generating 2 × 150 bp reads. Raw reads were evaluated with FastQC v0.12.1 (8). A total of 23,917,271 paired-end read pairs were obtained, with 97.3% of bases showing Q30 or higher quality scores. FastQC detected no adapter contamination or overrepresented sequences; therefore, no trimming was performed. Consequently, all 23,917,271 paired-end read pairs were retained for genome assembly.

*De novo* assembly was performed using SPAdes v3.15.0 (9) in careful mode with k-mers 21, 33, 55, 77, 99, and 127 and automatic coverage cutoff. SPAdes was selected based on the high quality and depth of the Illumina data set. Assembly statistics evaluated with QUAST v5.3.0(10) showed a 53.0 Mb genome distributed across 1,750 scaffolds, with the largest contig of 1.91 Mb and scaffold N50 of 441.4 kb.

Genome completeness was assessed using BUSCO v5.8.0 (11) with the hypocreales_odb10 data set (*n* = 4,494), revealing 99.0% completeness, 98.6% single-copy, and 0.4% duplicated genes, indicating a highly complete genome. Average nucleotide identity (ANI) analysis was performed using FastANI v1.3 (12) implemented in Galaxy (13), with default parameters, showing 93.47% similarity to the reference genome *R. rugulosa* CBS126565 (GCA_023509875.1).

Gene prediction was performed using AUGUSTUS v3.5.0 (14) implemented in BUSCO v5.8.0 (11), identifying 20,816 predicted protein-coding genes. Functional annotation with InterProScan v5.59-91.0 (15) and dbCAN3 (16) identified 618 carbohydrate-active enzymes distributed across 156 CAZyme families. Secondary metabolite analysis using antiSMASH/fungiSMASH v6.0 (17) identified 71 biosynthetic gene clusters.

PHI-base v4.18 (18) analysis was performed using DIAMOND BLASTp v2.1.22 (19) in sensitive mode with an e-value cutoff of ≤1 × 10⁻⁵, ≥30% amino acid identity, and ≥50% query coverage identified 1,395 homologs associated with pathogen-host interaction phenotypes (18). Additional assembly and functional annotation statistics are summarized in Table 1.

**TABLE 1:** Genome assembly, annotation, and functional features of *R. rugulosa* strain M7C8, including the distribution of CAZyme families, secondary metabolite biosynthetic gene clusters, and PHI-base pathogenicity-related gene categories

Category	Feature	Value
Sequencing	Sequencing platform	Illumina NovaSeq
	Library preparation	TruSeq Nano DNA kit
	Read type	Paired-end (2 × 150 bp)
	Number of reads	23,917,271
	Q30 bases	97.3%
Assembly statistics	Assembler	SPAdes v3.15.0
	Assembly mode	careful
	Genome size	53.0 Mb
	Number of scaffolds	1,750
	Largest contig	1.91 Mb
	Scaffold N50	441.4 kb
	GC content	52.87%
Genome completeness	BUSCO completeness	99.0%
	Single-copy BUSCOs	98.6%
	Duplicated BUSCOs	0.4%
	Fragmented BUSCOs	0.5%
	Missing BUSCOs	0.5%
Gene prediction	Predicted protein-coding genes	20,816
CAZyme annotation	Total CAZymes	618
	Most abundant CAZyme families	AA1 (14), AA7 (12), AA3 (11), GH13 (9), GH43 (9)
Secondary metabolism	Total biosynthetic gene clusters	71
	Terpene clusters	17
	Type I PKS clusters	16
	NRPS clusters	14
	Other cluster types	NRPS-like, betalactone, indole, phosphonate, isocyanide
Pathogenicity-related genes	Total PHI-base homologs	1,395
	Reduced virulence	769
	Unaffected pathogenicity	462
	Loss of pathogenicity	79
	Hypervirulence	59
	Lethal phenotypes	26
Comparative genomics	ANI similarity to *R. rugulosa* CBS126565	93.47%

## Data Availability

Raw sequencing reads generated using the Illumina NovaSeq 6000 platform for Rugonectria rugulosa strain M7C8 have been deposited in the NCBI Sequence Read Archive (SRA) under accession number SRR38177893. All records are associated with BioProject PRJNA1418200 and BioSample SAMN55040463.
